# Analysis of yellow mutant rainbow trout transcriptomes at different developmental stages reveals dynamic regulation of skin pigmentation genes

**DOI:** 10.1038/s41598-021-04255-y

**Published:** 2022-01-07

**Authors:** Shenji Wu, Jinqiang Huang, Yongjuan Li, Lu Zhao, Zhe Liu

**Affiliations:** 1 College of Animal Science and Technology, Gansu Agricultural University, Lanzhou, 730070 China; 2 College of Science, Gansu Agricultural University, Lanzhou, 730070 China

**Keywords:** Developmental biology, Genetics, Molecular biology

## Abstract

Yellow mutant rainbow trout (YR), an economically important aquaculture species, is popular among consumers due to its excellent meat quality and attractive appearance. Skin color is a key economic trait for YR, but little is known about the molecular mechanism of skin color development. In this study, YR skin transcriptomes were analyzed to explore temporal expression patterns of pigmentation-related genes in three different stages of skin color development. In total, 16,590, 16,682, and 5619 genes were differentially expressed between fish at 1 day post-hatching (YR1d) and YR45d, YR1d and YR90d, and YR45d and YR90d. Numerous differentially expressed genes (DEGs) associated with pigmentation were identified, and almost all of them involved in pteridine and carotenoid synthesis were significantly upregulated in YR45d and YR90d compared to YR1d, including *GCH1*, *PTS*, *QDPR*, *CSFIR1*, *SLC2A11*, *SCARB1*, *DGAT2*, *PNPLA2*, *APOD*, and *BCO2*. Interestingly, many DEGs enriched in melanin synthesis pathways were also significantly upregulated, including melanogenesis (*MITF*, *MC1R*, *SLC45A2*, *OCA2*, and *GPR143*), tyrosine metabolism (*TYR*, *TYRP1*, and *DCT*), and MAPK signaling (*KITA*) pathways. Using short time-series expression miner, we identified eight differential gene expression pattern profiles, and DEGs in profile 7 were associated with skin pigmentation. Protein–protein interaction network analysis showed that two modules were related to xanthophores and melanophores. In addition, 1,812,329 simple sequence repeats and 2,011,334 single-nucleotide polymorphisms were discovered. The results enhance our understanding of the molecular mechanism underlying skin pigmentation in YR, and could accelerate the molecular breeding of fish species with valuable skin color traits and will likely be highly informative for developing new therapeutic approaches to treat pigmentation disorders and melanoma.

## Introduction

Skin colors and patterns of fish are striking and important economic traits. Surface pigmentation, the basis for trait diversification, occurs in all animals from invertebrates to mammals, and is the result of fitness under natural conditions. Investigating this phenomenon offers an excellent opportunity to understand key issues in evolutionary biology such as adaptation, speciation, and maintenance of balanced polymorphisms^1,2^. Coloration is determined mainly by diverse pigments synthesized by pigment cells that are developed and differentiated from neural crest cells during embryonic development^3^. Unlike mammals, in which only melanophores (black or brown) are present, and amphibians and reptiles which possess xanthophores (yellow), erythrophores (red or orange), and iridophores (reflecting), six types of pigment cells, namely melanophores, xanthophores, erythrophores, iridophores, leucophores (white), and cyanophores (blue), have been identified in teleosts based on their hue, implying great plasticity in skin coloration^4^. Owing to the diversity of pigment cells in fish, skin color has become an essential phenotypic characteristic that assists the breeding of high yield and superior quality, and helps increase economic value^5^. In addition, visible coloration polymorphisms provide a traceable system within which to examine the molecular basis of adaptation and evolution owing to simple patterns of inheritance and the general ease with which morph/allele frequencies can be estimated^6^.

Due to the considerable conservation of pigmentation-related genes between teleosts and mammals^7^, the genetic mechanism of pigment production and subsequent skin pigmentation in teleosts has attracted the attention of many researchers. In recent decades, the field of vertebrate pigmentation has benefited greatly from model fish species including zebrafish (*Danio rerio*) and medaka (*Oryzias latipes*); genes including those in the tyrosinase gene family (*TYR*, *TYRP1*, and *DCT*), microphtalmia-associated transcription factor (*MITF*), melanocortin 1 receptor (*MC1R*), GTP cyclohydrolase 1 (*GCH1*), solute carrier family 2 member 11 (*SLC2A11*), solute carrier family 7 member 11 (*SLC7A11*), SRY-box 5 (*SOX5*), and colony-stimulating factor 1 receptor 1 (*CSF1R1*) are involved in melanogenesis and pteridine synthesis pathways, and they have been well studied^8–10^. RNA sequencing (RNA-seq) has proven to be a valuable tool for exploring gene and pathway responses, including the molecular mechanism of skin color variation in non-model fish species. Significant research has been undertaken on species with two or three different skin colors, examples of which include common carp (*Cyprinus carpio*)^9^, crucian carp (*Carassius carassius*)^11^, catfish (*Pelteobagrus fulvidraco*)^12^, and pufferfish (*Takifugu obscurus*)^13^. However, only a handful of studies have focused on the identification and characterization of specific genes involved in the pigmentation process in fish. Research in this could more clearly define the roles of genes related to specific pigments. For instance, Tian et al.^4^ found that elevated expression of carotenoid/pteridine metabolism genes including *GCH1*, *CSF1*, xanthine dehydrogenase (*XDH*), pair box 7 (*PAX7*), and scavenger receptor class B member 1 (*SCARB1*) were associated with the gradual formation of yellow skin in Japanese ornamental carp (*Cyprinus carpio* var. Koi). In a study by Zhang et al.^14^, melanogenesis genes (tyrosinase gene family members, *MITF*, *MC1R*, etc.) were found to be significantly downregulated in the gray-to-red transition in crucian carp (*Carassius auratus*, red var.), whereas *GCH1*, *XDH* and pyruvoyl tetrahydrobiopterin synthase (*PTS*) were significantly upregulated. Nevertheless, the molecular mechanism of pigmentation in most non-model fish remains poorly understood.

Yellow mutant rainbow trout (*Oncorhynchus mykiss*, YR), a major commercial and cold water fish species worldwide, is popular with consumers due to its excellent meat quality and attractive appearance. Several studies have explored the coloring characteristics of different phenotypes in this species. For example, Hattori et al.^15^ reported that 2-year-old YR individuals have a large number of xanthophores but no melanophores. Therefore, we believe that this color variation is due to a lack of melanin pigmentation in skin, just like albinism reported in other species. Then an interesting issue is to address what is the molecular mechanism governing skin color development in YR. In the present study, RNA-seq was conducted to identify candidate genes responsible for skin color development in YR during three separate developmental stages. Several candidate genes and important pathways were identified that might regulate skin color formation in YR skin. The results deepen our understanding of the molecular mechanism underlying pigmentation in YR, and could accelerate the molecular breeding of fish species with valuable skin color traits and will likely be highly informative for developing new therapeutic approaches to treat pigmentation disorders and melanoma.

## Results

### Overview of RNA-seq data

**Figure 1:**
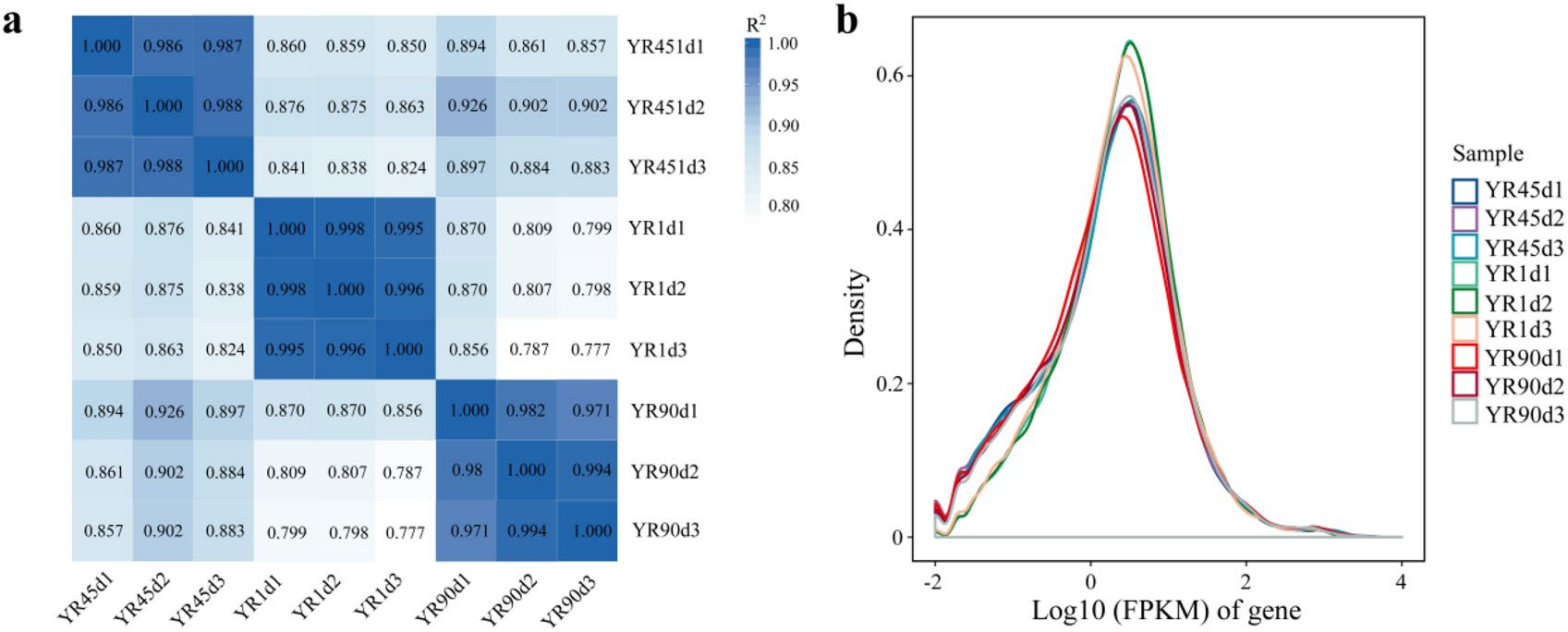
(**a**) Heatmap of Pearson correlation between samples. The number in the boxes refer to Pearson’s correlation coefficients (R^2^), and the closer R^2^ is to 1, the higher degree of correlation between samples. (**b**) Distribution of gene expression abundance of each sample. The x-axis represents the values of log10 (FPKM) of gene, the y-axis represents the number of genes corresponding to the value of the x-axis/the total number of genes.

To identify skin pigmentation-related genes that are differentially expressed at different developmental stages of YR, three ontogenetic stages (1 day post-hatching (1 dph), 45 dph, and 90 dph) were selected to conduct a comparative transcriptome analysis. In total, 151,386,318, 151,800,162, and 140,467,856 raw reads were obtained from YR1d, YR45d, and YR90d groups, respectively, and were deposited at the National Center for Biotechnology Information (NCBI) database under accession number GSE179976. After filtering ambiguous nucleotides and low-quality sequences, 150,328,264, 151,116,680, and 139,543,156 clean reads were obtained. An overview of quality control is shown in Table 1; Q20% was 96.98–98.22%, Q30% was 92.08–94.95%, and GC% was 47.64–52.62%. The unique ratio of mapping to the rainbow trout genome ranged from 86.43 to 87.51% (Table 1). Pearson’s correlation coefficients (R^2^) for sample expression were 99.5–99.8% for YR1d1, YR1d2, and YR1d3, 98.6–98.8% for YR45d1, YR45d2, and YR45d3, and 97.1–99.4% for YR90d1, YR90d2, and YR90d3, confirming the robustness of the biological replicates and the reliability of the RNA-seq results (Fig. 1a). These results showed that the sequencing data generated in this study were reliable and could be used for further analysis.

**Table 1:** Summary statistics of the transcriptome.

Sample ID	Raw reads	Clean reads	Q30 (%)	GC content (%)	Total mapped	Uniquely mapped
YR1d1	49,915,264	49,555,578	92.11	48.13	45,858,022 (92.66%)	42,773,682 (86.43%)
YR1d2	50,049,856	49,674,724	92.08	48.21	45,998,957 (92.76%)	42,896,366 (86.50%)
YR1d3	514,211,98	51,097,962	92.23	47.64	47,378,160 (92.86%)	44,226,983 (86.69%)
YR45d1	458,784,40	45,676,770	93.49	52.51	43,069,518 (94.80%)	39,636,571 (87.25%)
YR45d2	542,814,14	54,027,926	93.33	52.62	49,575,889 (94.57%)	45,658,960 (87.10%)
YR45d3	516,403,08	51,411,984	93.48	52.49	46,750,406 (94.77%)	43,065,342 (87.30%)
YR90d1	50,249,522	49,906,702	94.95	48.51	46,541,643 (93.39%)	43,611,292 (87.51%)
YR90d2	45,786,898	45,465,258	94.90	48.05	42,168,630 (92.91%)	39,625,882 (87.31%)
YR90d3	44,431,436	44,171,196	94.95	47.91	40,833,717 (92.56%)	38,338,573 (86.90%)

**Table 2:** Primers used for qRT-PCR of selected genes.

Gene_ID	Gene name	Forward primer sequences (5′–3′)	Reverse primer sequences (5′–3′)
110502330	*GCH1*	TTGAAAGAGCCTACGGCATCG	TGGCTGCCCGTAATGGTGT
110525343	*SLC2A11*	TTGCCTTACTGGCTGCTCTGT	GCCCGCATTTACTCCTGTGA
110504828	*CSF1R1*	AATGGCGGCATCCTGTAATC	CTAATGGGGTGCCTGGGTT
110523398	*SCARB1*	TTTATCTCCCACCCGCACTT	ACCAGTCTCAGGGTGGATGTCT
110537459	*DGAT2*	CGCAGTTCTTCAGTGGGTCA	TGATGAGCCTGATGGGAAAGT
110490314	*PNPLA2*	GACGCCCTTCGCTTTCTTG	TTGATTTGGGTTGGCAGGTT
100135828	*TYR*	ATTCTTGGGTCATTCTGTTGGC	ATTCAAGTGACGCACCCGAC
110500604	*TYRP1*	ATGGCTTGCTGGTGCTCTTG	TGACCACTCCGAAGTCCCTCT
110520164	*DCT*	ACGAGGAAACTGCTCCGACAT	CCACCAAAGTTACCTGAGCACC
110494592	*MITF*	ATGGTCTTGCGGTTGTGCC	GTGGATGGTGCCGTTGTTGA
110505140	*QDPR*	GTAACCTTGGATACGCCGATGA	CTATTGCGTGGGTAGTGTCTGTG
100302645	*SOX5*	GGCTGTTGTCTAAGGACTGGAAG	ATCTGCTGCCGCTGCTTCT
110508192	*PAX3*	AGCAAACCGAAGACATCCACC	ATCGTCCTCCTCGTCTTCTCC
110505014	*SLC7A11*	TGTGGAGTGCCCGAGATTG	ATTCTCAAAGTTCTTCGTCTCCC

### Differential expression analysis

**Figure 2:**
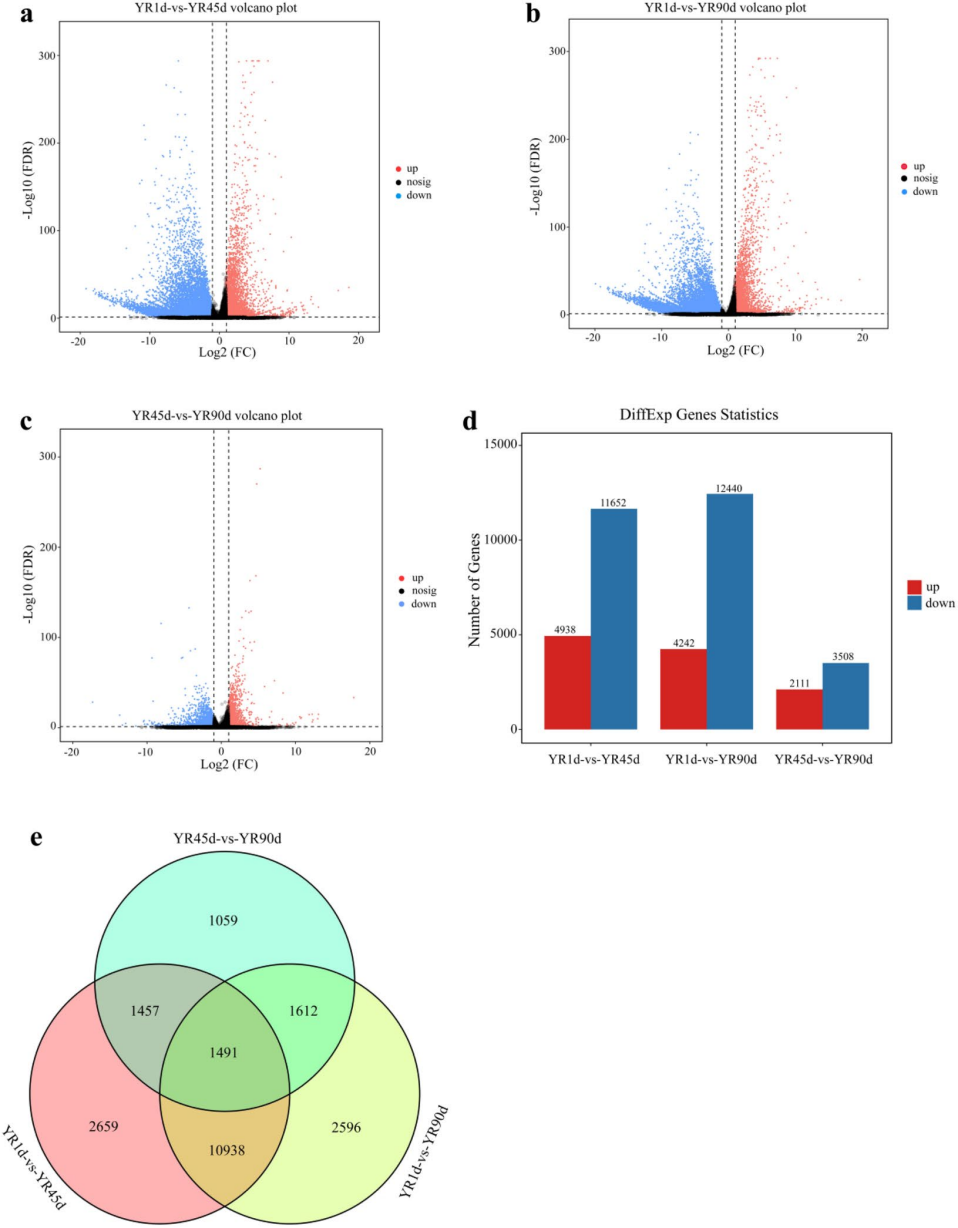
Distributions of differentially expressed genes (DEGs) and comparative results of RNA-seq among the three compared groups (YR1d vs. YR45d, YR1d vs. YR90d, YR45d vs. YR90d). (**a**–**c**) showed volcano scatter plot of DEGs among the three compared groups, respectively. (**d**) Numbers of upregulated and downregulated DEGs among the three compared groups. (**e**) Visualization of the DEGs in a venn diagram indicated that 1491 DEGs were shared among the three compared groups.

**Figure 3:**
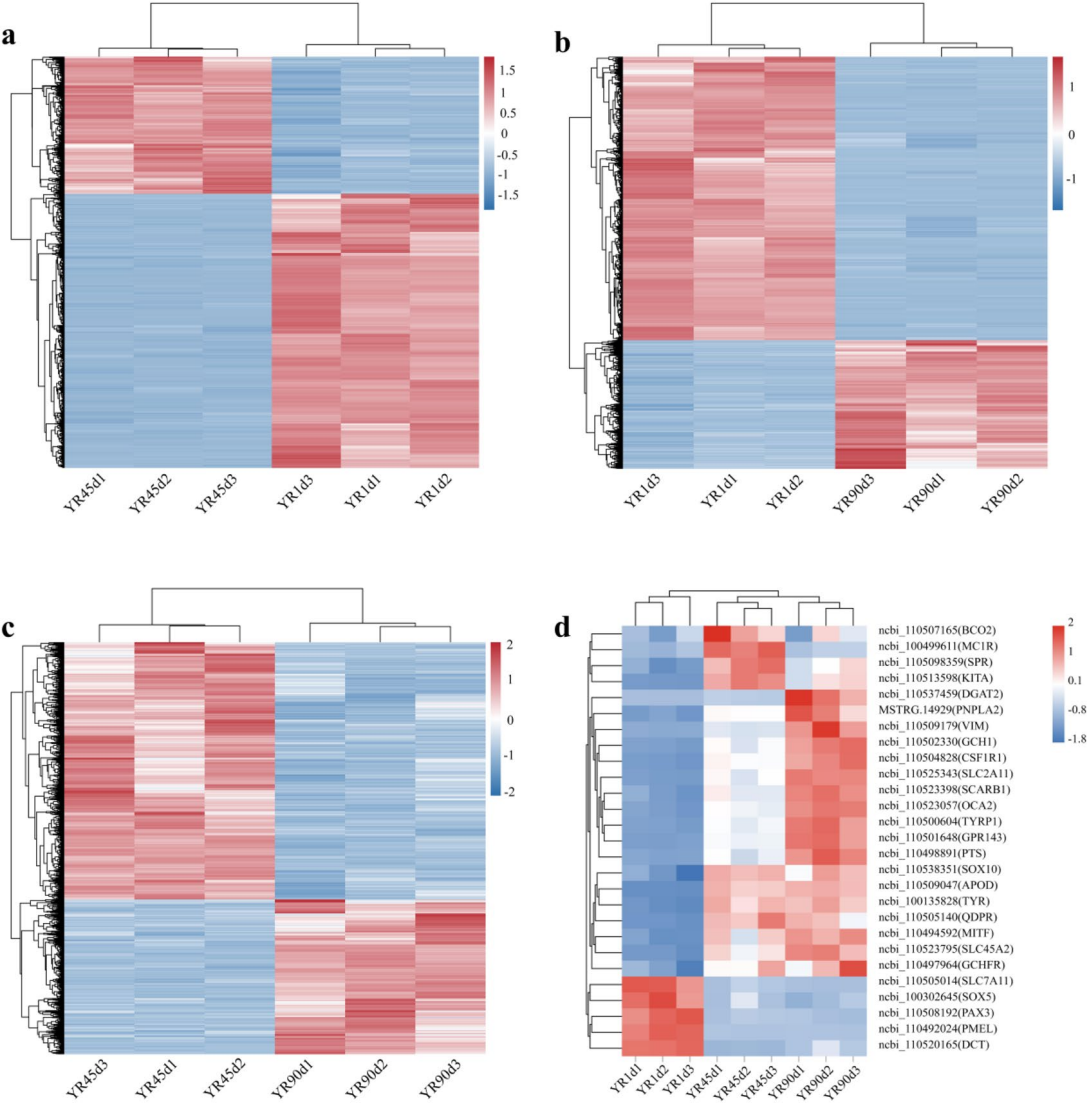
Heatmaps of expression levels of all DEGs and pigmentation-related genes among the three compared groups, representing three biological replicates. (**a**) YR1d vs. YR45d. (**b**) YR1d vs. YR90d. (**c**) YR45d vs. YR90d. (**d**) Pigmentation-related genes.

An identical number of genes (43,862) was identified by performing a pairwise comparison among the three groups, including 39,862 known and 4000 novel genes. Using fragments per kilobase per million (FPKM) to normalize gene expression levels, the distribution of gene expression abundance was assessed for each sample (Fig. 1b). Based on |log2 fold-change| > 1 and false discovery rate (FDR) < 0.05 as thresholds, 16,590, 16,682, and 5619 differentially expressed genes (DEGs) were identified for YR1d vs. YR45d, YR1d vs. YR90d, and YR45d vs. YR90d comparisons. Of these DEGs, 4938, 4242, and 2111 were upregulated and 11,652, 12,440, and 3508 were downregulated, respectively (Fig. 2a–d). Further information can be found in Table S1. Intersection analysis for the different comparisons revealed 12,429 DEGs shared between YR1d vs. YR45d and YR1d vs. YR90d, and 1491 genes were shared among the three compared groups (Fig. 2e). Additionally, heatmaps were generated to reveal variable expression patterns for all DEGs and skin pigmentation-related genes among the three compared groups (Fig. 3).

**Figure 4:**
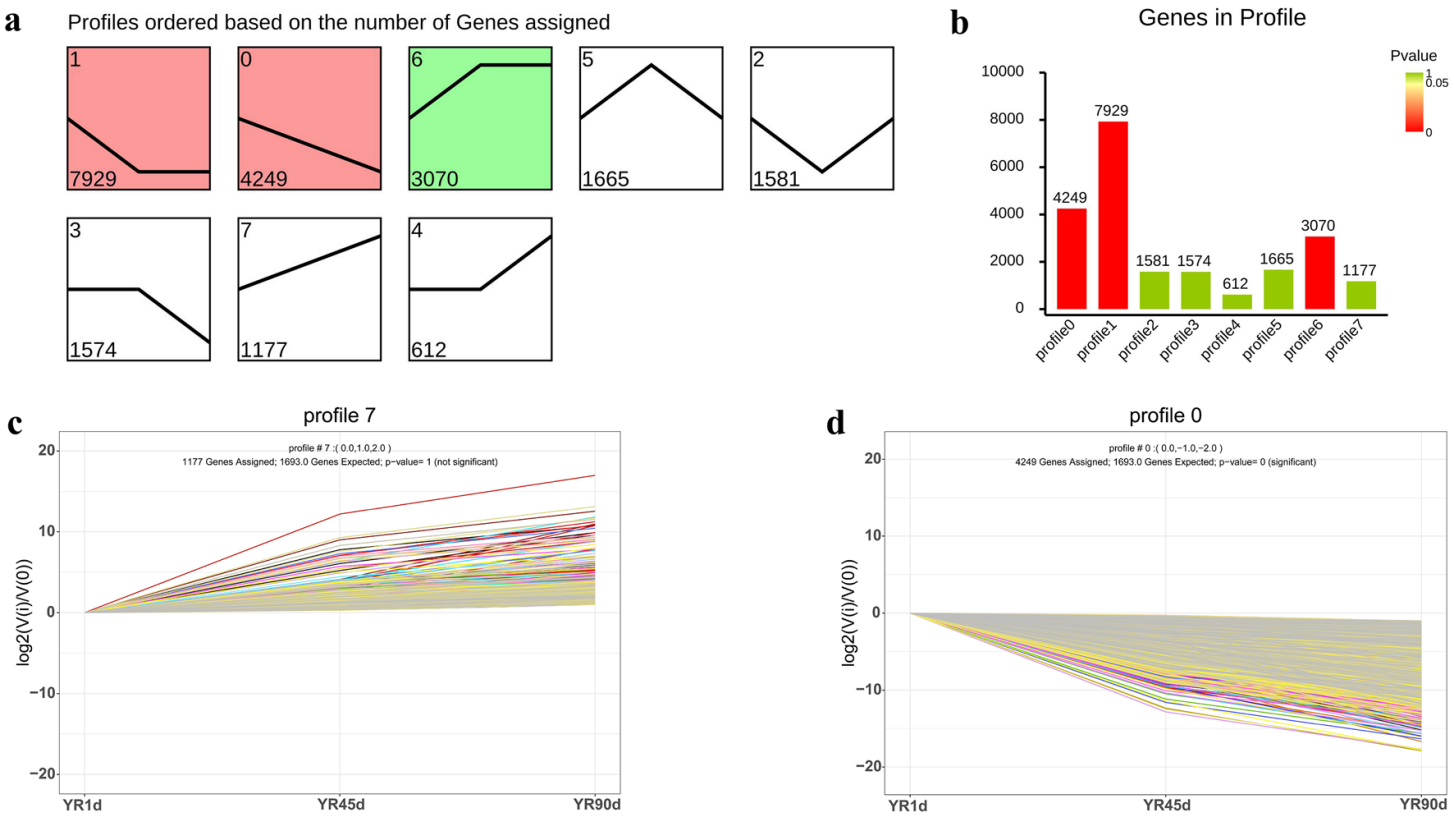
Trend analysis for DEGs. (**a**) Profiles of 8 differently expressed mRNAs. (**b**) Numbers of DEGs in each profile. (**c**) Details of profile 7 in which the expression of genes were continuously upregulated from 1 to 90 dph. (**d**) Details of profile 0 in which the expression of genes were continuously downregulated from 1 to 90 dph.

In order to understand the dynamic changes of DEGs in different developmental stages of YR, DEGs were subjected to expression trend analysis using short time-series expression miner (STEM). Based on the results, DEGs were divided into eight profiles, and most were enriched in profiles 1 and 0 (Fig. 4a,b). In profile 7, expression of genes was continuously upregulated from 1 to 90 dph (Fig. 4c). By contrast, expression of genes was continuously downregulated from 1 to 90 dph in profile 0 (Fig. 4d). Among the continuously upregulated DEGs related to pigments were *GCH1*, *SLC2A11*, *SCARB1*, *DGAT2*, *GCHFR*, *PTS*, *PAPLA2*, *VIM*, *CSF1R1*, *APOD*, *TYR*, *TYRP1*, *MITF*, *SLC45A2*, *OCA2*, and *GPR143*. Among the continuously downregulated DEGs related to pigments were *SOX5*, *PAX3*, and *SLC2711*. These pigment-specific genes are closely associated with pteridine/carotenoid/melanin synthesis. The overall expression levels of these pigment-specific genes were higher in YR45d and YR90d than YR1d, however, most did not differ significantly between YR45d and YR90d. Detailed information is included in Table 3.

**Table 3:** Representative pigmentation-related genes differentially expressed in each compared groups.

Gene name	Description	YR1d-vs-YR45d		YR1d-vs-YR90d		YR45d-vs-YR90d	
		Log2 (FC)	FDR	Log2 (FC)	FDR	Log2 (FC)	FDR
**Pteridine synthesis**							
*GCH1*	GTP cyclohydrolase 1	2.27	1.18E − 68	3.17	4.60E − 165	0.89	6.31E − 12
*SLC2A11*	Solute carrier family 2 member 11	2.44	2.78E − 48	3.30	4.08E − 110	0.86	6.15E − 09
*CSF1R1*	Colony-stimulating factor 1 receptor 1	2.75	5.38E − 92	3.48	4.77E − 123	0.73	1.12E − 12
*PTS*	Pyruvoyl tetrahydrobiopterin synthase	1.16	1.67E − 14	2.00	1.76E − 45	0.83	2.11E − 07
*QDPR*	Dihydropteridine reductase	2.14	3.37E − 70	1.94	8.60E − 33	− 0.21	0.78
*SOX5*	Transcription factor sox-5	− 1.87	3.34E − 06	− 2.40	4.28E − 08	− 0.52	0.54
*PAX3*	Paired box protein pax-3	− 3.97	3.10E − 32	− 5.08	3.10E − 23	− 1.39	0.32
**Carotenoid synthesis**							
*SCARB1*	Scavenger receptor class B member 1	2.07	2.27E − 16	2.96	6.16E − 32	0.89	2.82E − 08
*DGAT2*	Diacylglycerol O-acyltransferase 2	3.50	6.98E − 52	6.78	9.27E − 152	3.29	9.50E − 55
*PNPLA2*	Patatin-like phospholipase protein 2	3.68	6.77E − 11	4.42	2.26E − 15	0.74	0.02
*APOD*	Apolipoprotein D	5.58	0.00	5.68	0.00	0.10	0.16
*BCO2*	Beta,beta-carotene 9′,10′-oxygenase	1.69	0.01	2.35	8.21 × 10^−4^	− 1.04	0.31
**Melanin synthesis**							
*TYR*	Tyrosinase precursor	1.84	1.14E − 62	1.86	1.43E − 56	0.03	0.43
*TYRP1*	Tyrosinase-related protein 1	1.86	1.40E − 89	2.72	3.67E − 121	0.86	4.78E − 12
*DCT*	l-Dopachrome tautomerase	3.31	2.04E − 95	4.17	1.69E − 136	0.86	7.76E − 14
*MITF*	Microphthalmia transcription factor	1.62	4.82E − 14	1.99	4.38E − 34	0.36	0.09
*MC1R*	Melanocortin 1 receptor	2.25	7.36E − 35	1.70	3.87E − 12	− 0.55	0.10
*PMEL*	Melanocyte protein	− 6.02	5.21E − 122	− 6.67	4.13E − 93	− 0.64	0.61
*SLC45A2*	Solute carrier family 45 member 2	1.80	2.58E − 37	2.23	2.97E − 49	0.43	3.17 × 10^−3^
*OCA2*	P protein	2.10	6.43E − 37	2.96	7.23E − 73	0.86	1.50E − 11
*GPR143*	G-protein coupled receptor 143	2.03	2.05E − 29	2.91	2.56E − 55	0.88	2.95E − 08
*KITA*	Stem cell growth factor receptor kita	3.22	3.23E − 65	2.60	2.16E − 27	− 0.62	0.02
*SLC7A11*	Cystine/glutamate transporter	− 3.92	2.16E − 14	− 6.60	5.69E − 11	− 2.67	0.17

### Gene Ontology (GO) enrichment and Kyoto Encyclopedia of Genes and Genomes (KEGG) pathway analyses

**Figure 5:**
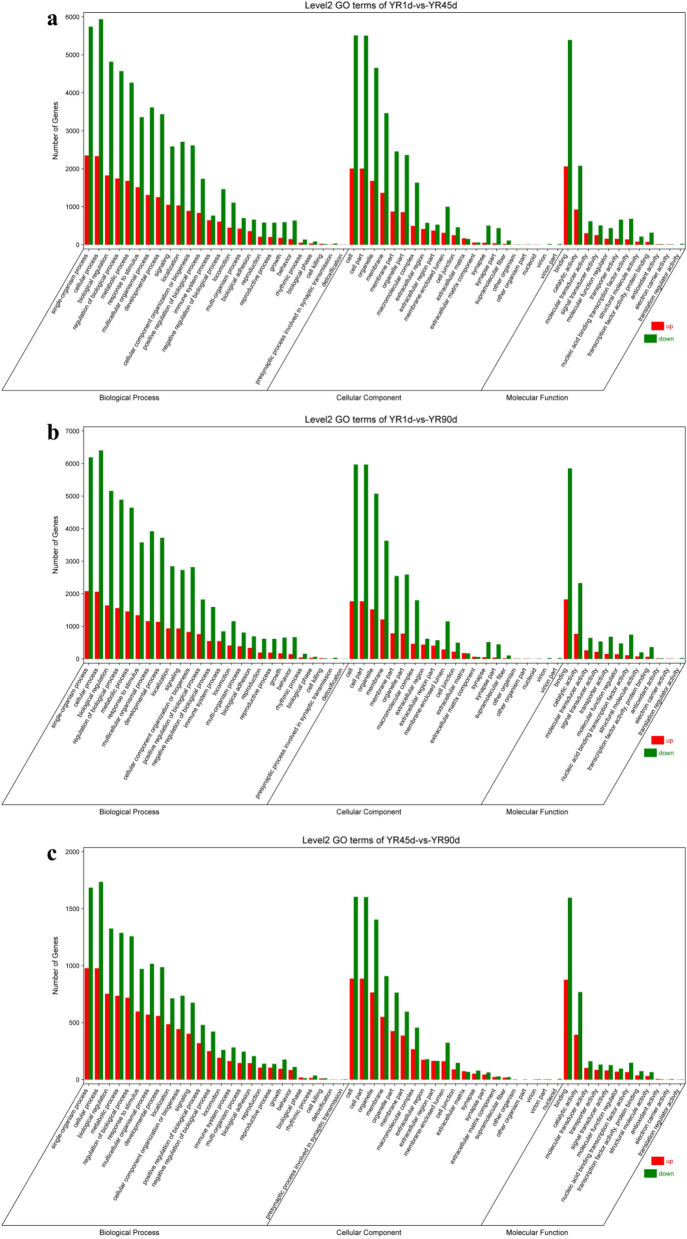
GO categorization of DEGs among the three compared groups. (**a**) YR1d vs. YR45d. (**b**) YR1d vs. YR90d. (**c**) YR45d vs. YR90d.

**Figure 6:**
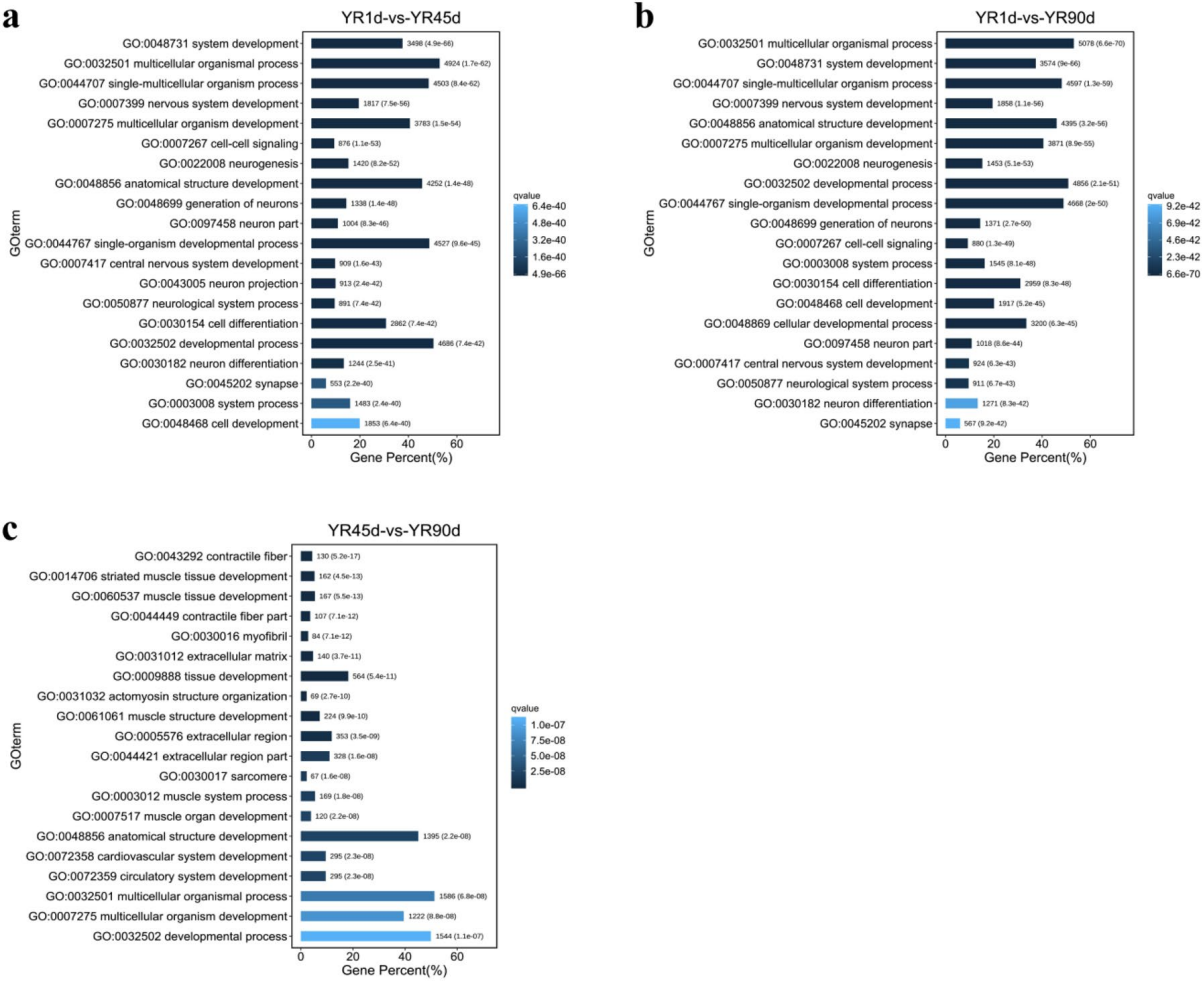
The top 20 GO terms among among the three compared groups. (**a**) YR1d vs. YR45d. (**b**) YR1d vs. YR90d. (**c**) YR45d vs. YR90d. Gene percent (%): the number of DEG in each term/the total of DEG number.

To evaluate the different functions of DEGs, GO functional enrichment analysis was conducted using the GO database. GO enrichment analysis was divided into three major categories: biological process, cellular component, and molecular function. Among the three compared groups, ‘single-organism process’, ‘cellular process’, and ‘biological regulation’ contained the most DEGs in the biological process category; ‘cell’, ‘cell part’, and ‘organelle’ contained the most DEGs in the cellular component category; ‘binding’, ‘catalytic activity’, and ‘molecular transducer activity’ contained the most DEGs in the molecular function category (Fig. 5). In the YR1d vs. YR45d and YR1d vs. YR90d comparisons, several significantly enriched GO terms associated with the nervous system and cells were included among the top 20 biological process subcategories, including ‘nervous system development’, ‘central nervous system development’, ‘cell–cell signalling’, ‘cell differentiation’, ‘cell development’, and ‘cellular developmental process’, while none of these GO terms appeared among the top 20 biological process subcategories in the YR45d vs. YR90d comparison (Fig. 6 and Table S2).

**Figure 7:**
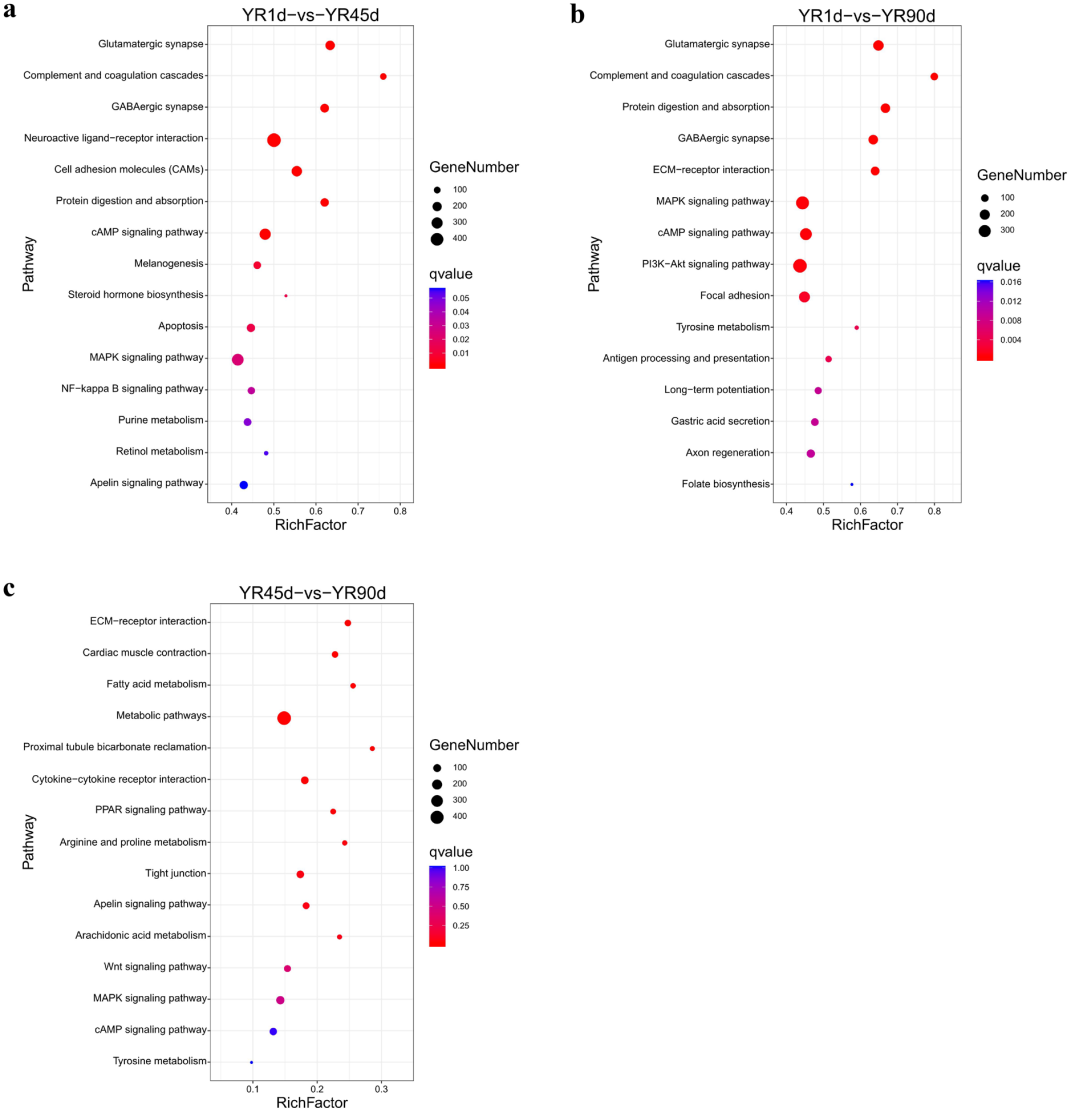
Scatterplot of enriched KEGG pathways (www.kegg.jp/kegg/kegg1.html) for DEGs among the three compared groups. (**a**) YR1d vs. YR45d. (**b**) YR1d vs. YR90d. (**c**) YR45d vs. YR90d. Rich factor is the ratio of the DEG number to the total gene number in a given pathway.

The transcriptome sequences were subsequently aligned to the KEGG database to identify biological pathways affected in the skin of YR in different developmental stages. In the YR1d vs. YR45d comparison, 68 pathways were significantly enriched (q-value < 0.05), and three enriched pathways related to pigment synthesis were identified: cAMP signaling pathway (305 DEGs), melanogenesis (130 DEGs), and MAPK signaling pathway (336 DEGs). In the YR1d vs. YR90d comparison, 55 pathways were significantly enriched (q-value < 0.05), and three enriched pathways were related to pigment synthesis: MAPK signaling pathway (359 DEGs), cAMP signaling pathway (288 DEGs), and tyrosine metabolism (36 DEGs). However, there were no significantly enriched pathways related to pigment synthesis in the YR45d vs. YR90d comparison (Fig. 7 and Table S3).

### Interaction network analysis of DEGs

**Figure 8:**
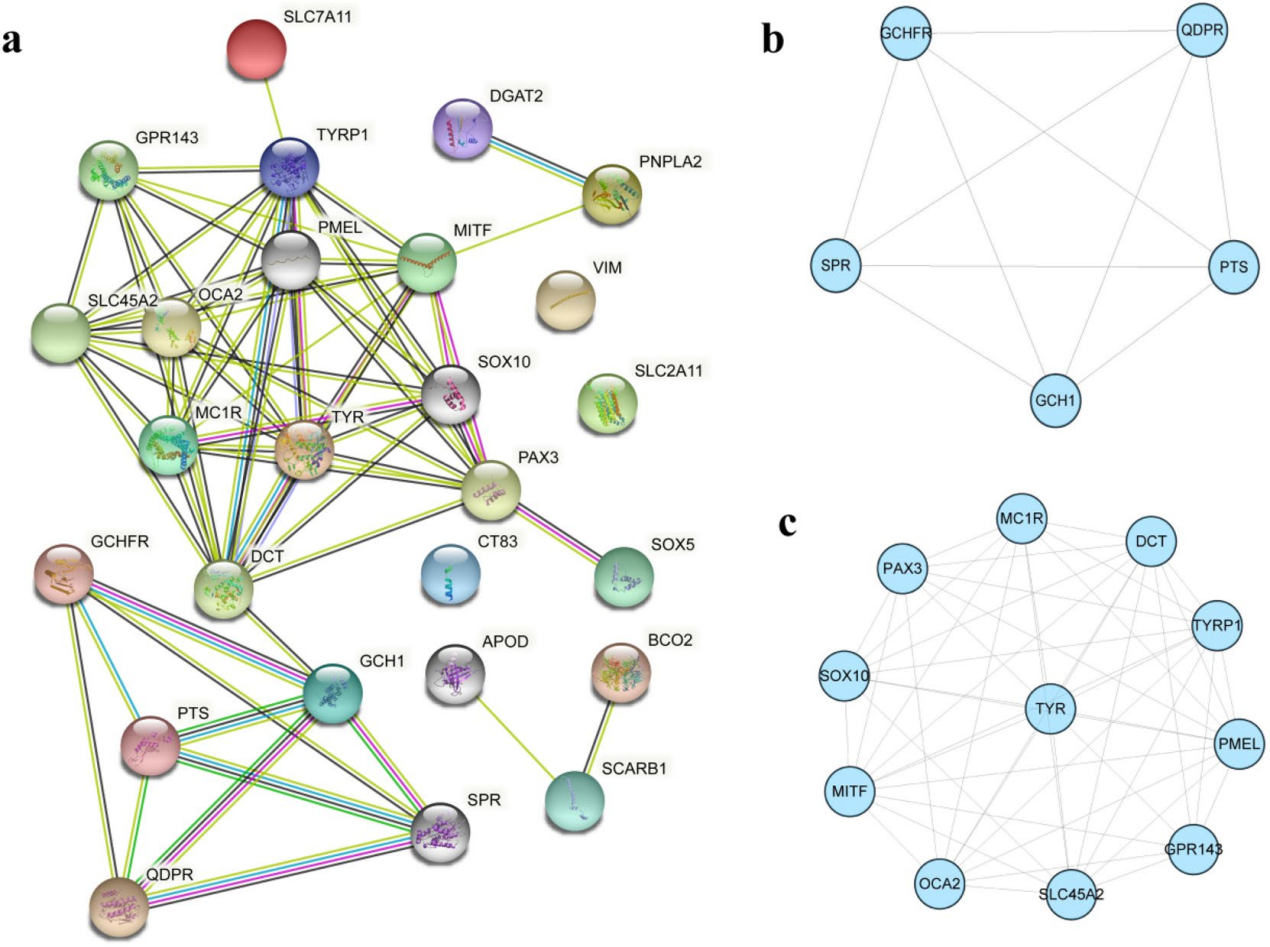
PPI network analysis of the pigmentation-related genes based on STRING database. (**a**) Overall PPI network of the pigmentation-related genes identified in this study. (**b**) Module associated with xanthophores. (**c**) Module associated with melanophores.

To gain insight into the biological significance of pigmentation-related genes identified in our study at the protein level, we constructed a protein–protein interaction (PPI) network of proteins encoded by the DEGs, resulting in 26 nodes and 67 edges (Fig. 8a). Additionally, two modules associated with xanthophores and melanophores were identified (Fig. 8b,c).

### Identification of simple sequence repeats (SSRs) and single-nucleotide polymorphisms (SNPs)

**Figure 9:**
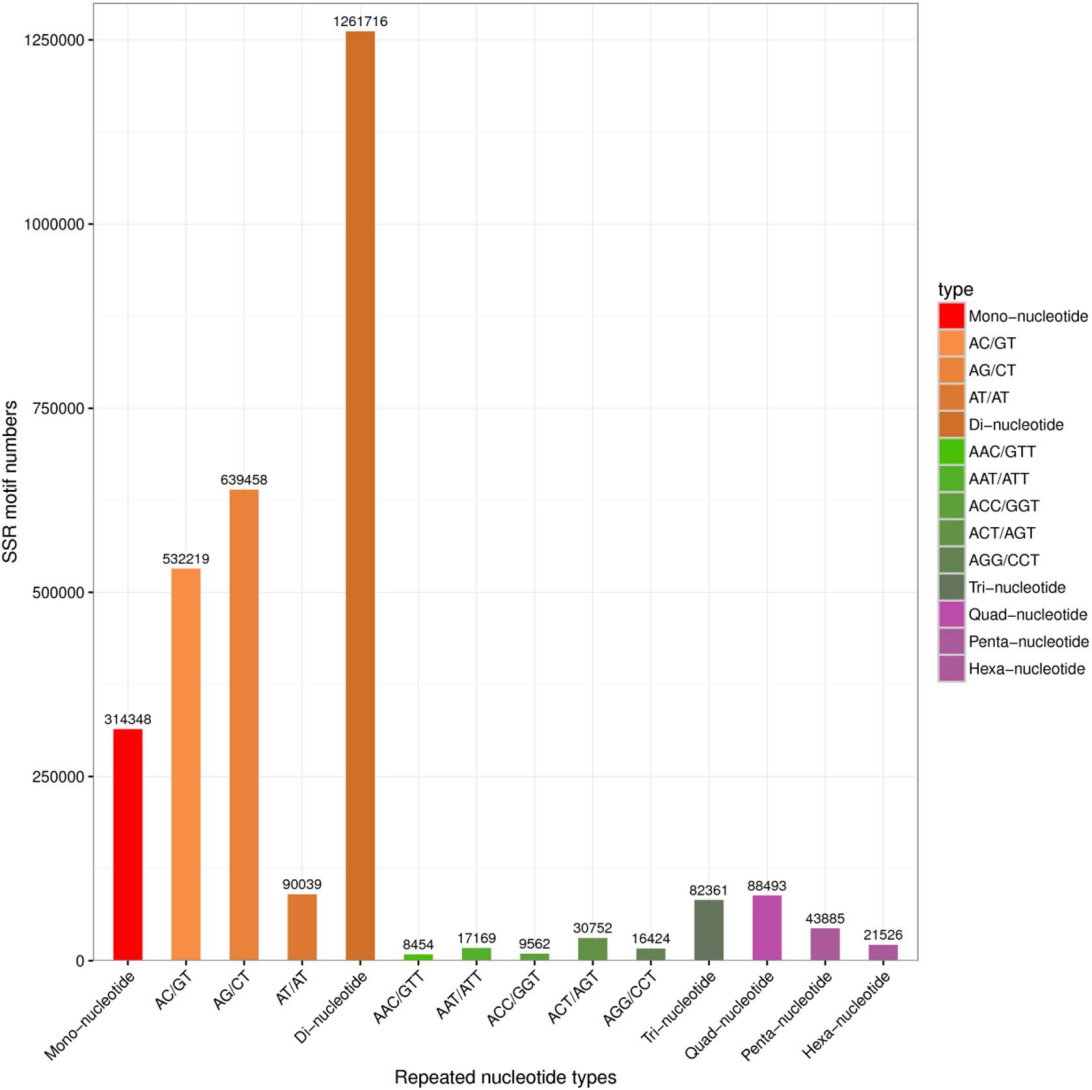
SSR motif repeat distribution in transcriptome data of YR.

In total, 1,812,329 SSRs were discovered in the YR transcriptomes. Among these SSRs, di-nucleotide motifs were the most abundant (1,261,716, 69.62%), followed by mono-nucleotide (314,348, 17.35%), tetra-nucleotide (88,493, 4.88%), tri-nucleotide (82,361, 4.54%), penta-nucleotide (43,885, 2.42%), and hexa-nucleotide (21,526, 1.19%) repeats. Among the di-nucleotide repeats, AG/CT (639,458) were the major types. The most and least common tri-nucleotides were ACT/AGT (30,752) and AAC/GTT (8454), respectively (Fig. 9).

**Figure 10:**
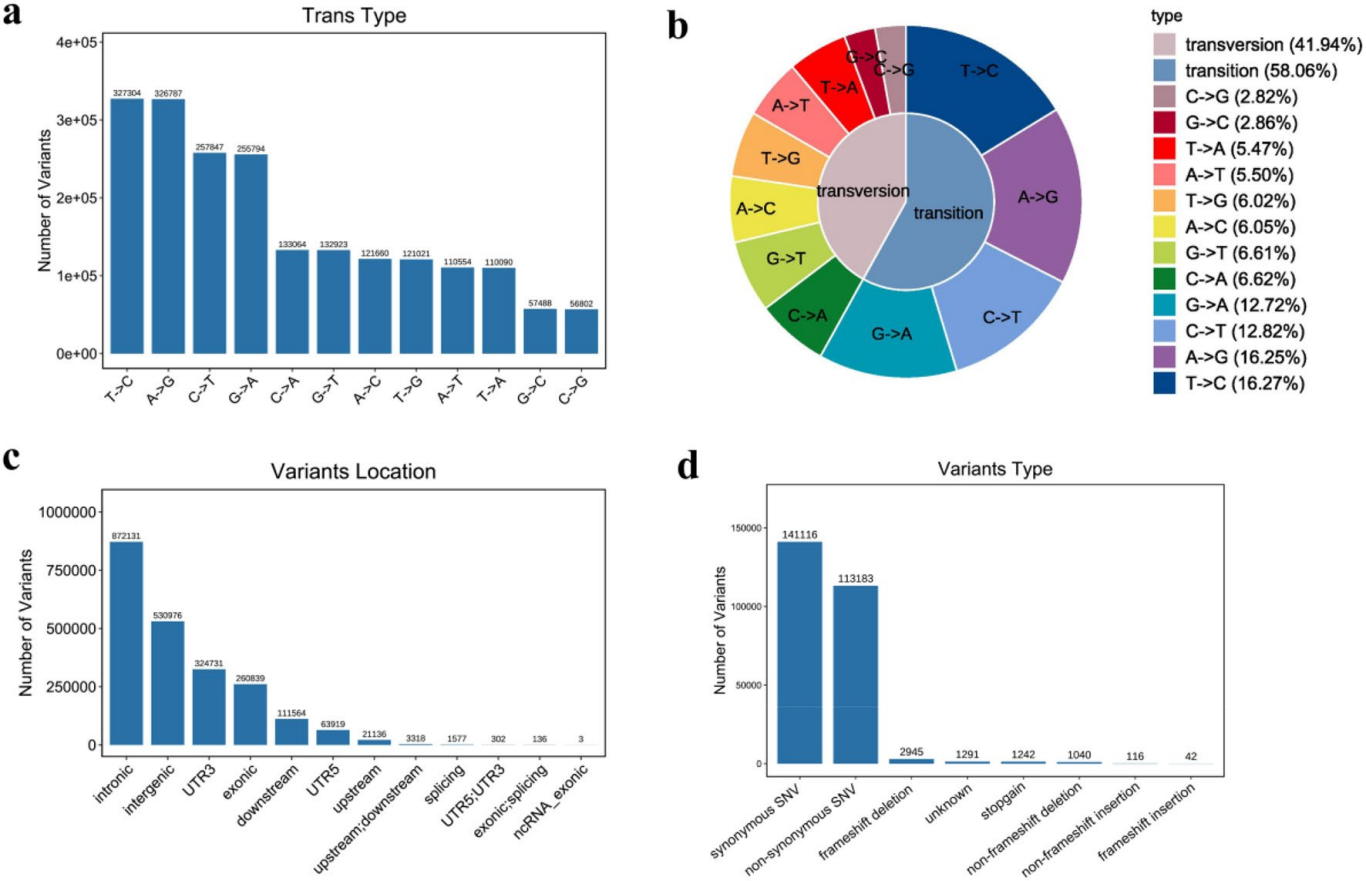
The type, location and function distributions of SNP. (**a**, **b**) Number and proportion of each type of SNP. (**c**) Location distribition of SNP. (**d**) Function classification of SNP.

A total of 2,011,334 SNPs were identified (Fig. 10a,b), among which 1,167,732 (58.06%) were transitions (A/G and C/T) and 843,602 (41.94%) were transversions (A/T, C/G, A/C and G/T). Most SNPs were located in intronic and intergenic regions (Fig. 10c), and most SNPs were synonymous mutations, followed by non-synonymous mutations (Fig. 10d).

### Validation of DEGs identified by RNA-Seq

**Figure 11:**
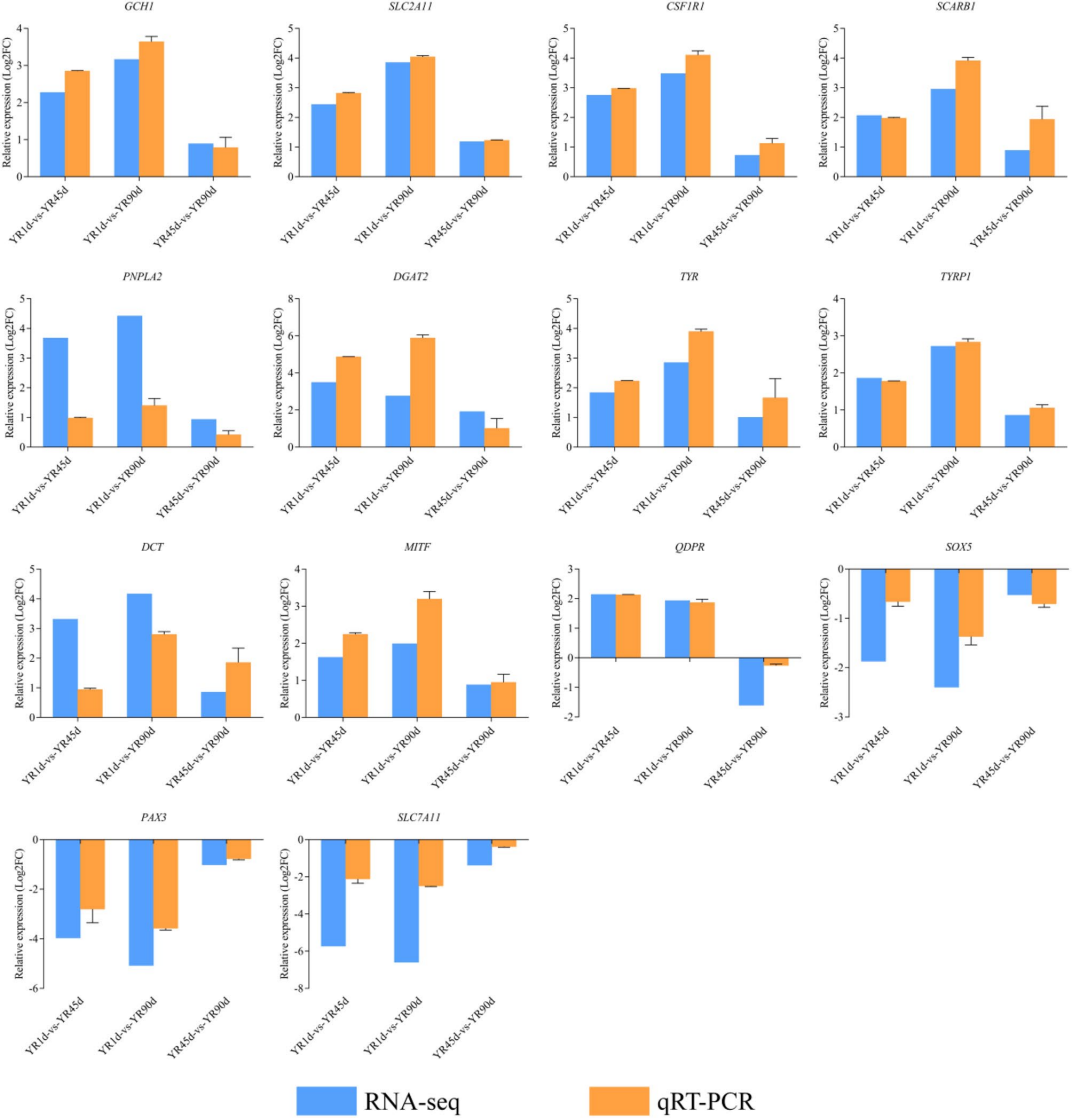
Validation of DEGs by qRT-PCR. The green columns indicate the results of RNA-seq and the red columns represent the results of qRT-PCR. Log2FC refers to the log2 fold change.

In order to confirm the reliability of DEGs identified by RNA-seq, we selected 14 DEGs for qRT-PCR analysis to determine their relative expression levels at different developmental stages (YR1d, YR45d, and YR90d). The qRT-PCR expression patterns were consistent with the RNA-seq results, confirming that the RNA-seq data were reliable (Fig. 11).

## Discussion

Skin color is an important economic trait for many farmed fish because it acts as a crucial criterion for visually determining quality and market value. Although the expression levels of pigment-specific genes are thought to be responsible for the great variety in skin pigmentation observed in model and ornamental fish^16^, the molecular mechanism of skin pigmentation in most non-model fish remains largely unknown. YR, a variant of rainbow trout and an economically important cold-water fish species, is an excellent model to explore the molecular mechanism that determines phenotype. In this study, we sought to identify genes involved in skin color formation by employing comparative transcriptome analyses among YR groups at three developmental stages (YR1d, YR45d, and YR90d). The results help us to understand the molecular mechanism of skin pigmentation in YR, and could accelerate the molecular breeding of fish species with valuable skin color traits and will likely be highly informative for developing new therapeutic approaches to treat pigmentation disorders and melanoma.

Chromatophores are responsible for skin coloration in fish, and their specification and differentiation into diverse pigment cell types is strictly regulated by cell type-specific gene expression profiles^14^. Besides melanophores, xanthophores with pteridine and carotenoid as coloring substances are also a common cell type in fish skin, and research revealed that differences in carotenoid and pteridine synthesis gene expression are correlated with differences in the visual appearance of yellow/red coloration^17,18^. In the present study, five pteridine synthesis-related genes (*GCH1*, *PTS*, *QDPR*, *CSFIR1*, and *SLC2A11*) were upregulated in YR45d and YR90d compared with YR1d. GCH1, the essential rate-limiting enzyme in pteridine synthesis, catalyzes the de novo synthesis of H4biopterin, a source of material for pteridine pigments in xanthophore precursors^18^. Although it is also reported to be involved in the process of melanogenesis by acting as an essential electron donor in phenylalanine hydroxylase-catalyzed conversion of l-phenylalanine into l-tyrosine, and for tyrosine hydroxylase isoform I-catalyzed generation of l-dihydroxyphenylalanine from l-tyrosine, the role of H4biopterin in pteridine pigment synthesis in fish has been more extentively studied to date^18–20^. In zebrafish, expression of *GCH1* increases as xanthophores develop during the embryonic stage^18^. Zhang et al.^11^ revealed that the expression level of *GCH1* in red skin of crucian carp was markedly higher than in black skin. PTS is a key cofactor for various enzymes in the pteridine synthesis pathway, including aromatic amino acid hydroxylases, and H4biopterin produced by GCH1 is converted into 6-pyruvoyltetrahydropterin by PTS^21,22^. In the case of inherited PTS deficiency, H4biopterin stores are depleted^21^. QDPR serves as another cofactor that is responsible for the regeneration of H4biopterin from quinoid dihydrobiopterin (qBH2)^23^. Another important gene is *CSFIR1*, which plays a critical role in development, proliferation, and dispersal of xanthophores^24,25^. *CSFIR1* can act cell-autonomously within xanthophores, and its mutation can almost eliminate xanthophores, leading to the absence of any orange colour in guppy (*Poecilia reticulate*)^25^. *SLC2A11* is known to be important in the development of xanthophores in teleosts by promoting their differentiation and yellow pigmentation^19,26^. The zebrafish mutation project found that an *SLC2All* mutant displayed a differentiation defect in its xanthophores^26^. As explained above, it is probable that these genes associated with the pteridine synthesis pathway might play important roles in the skin coloration of YR.

Another two candidate genes, *SOX5* and *PAX3*, were downregulated in YR45d and YR90d compared with YR1d. The protein SOX5 encoded by *ML-3* (many leucophores-3) is a member of the Sox family of transcription factors^27^. *SOX5* is required exclusively for the development of xanthophores, and loss of this gene leads to the complete absence of xanthophores^28^. Moreover, not only can *SOX5* autonomously regulate xanthophore specification, it can also contribute to enhance xanthophore lineages by antagonizing *SOX10*^29^. In a previous study, expression of *SOX5* was detected in neural crest and xanthophore precursors before the onset of *GCH* expression, and expression gradually decayed from 4 days post-fertilization (dpf) to 5 dpf^29^. As a marker of xanthophore lineage, *PAX3* (paired box gene 3) was previously thought to perform a conserved and fundamental role during neural crest development, but it was recently shown to be involved in the development of new populations of xanthophores^30,31^. Minchin et al.^32^ showed that knockdown of *PAX3* in zebrafish leads to the emergence of abnormal neural crest maintenance, and the number of xanthophores was drastically reduced and accompanied by an increase in melanophores. Herein, GO analysis revealed that *SOX5* and *PAX3* were significantly enriched in ‘central nervous system development’ and ‘pigment cell differentiation’ GO terms. Thus, upregulation of *SOX5* and *PAX3* in YR1d suggests that both may play a pivotal role in regulating the formation of xanthophore precursors.

Unlike pteridine, fish cannot synthesize carotenoids endogenously, and instead depend solely on uptake from their diet^33^. Due to their hydrophobic nature, carotenoids are not easily solubilized in the water environment of the gastrointestinal tract, hence processes involved in the gastrointestinal uptake and transport of carotenoid are closely associated with lipids^34^. Only a few genes known to influence carotenoid-based color diversity in vertebrates have been identified. SCARB1 is characterized as a high-density lipoprotein receptor that mediates the cellular uptake of carotenoids through binding to multiple ligands^35^. Kleveland et al.^36^ reported extremely high mRNA expression levels of *SCARB1* in the midgut of Atlantic salmon (*Salmo salar*). The first evidence that this gene plays a central role in carotenoid uptake was demonstrated in fruit fly (*Drosophila*), and this was subsequently confirmed in canary (*Serinus canaria*)^35,37^. Importantly, recent research found that *SCARB1* is indispensable for the deposition of carotenoids in adult xanthophores in zebrafish^38^. Following absorption, lipophilic carotenoids are converted to retinoids and other apocarotenoid metabolites via a series of enzymatic reactions^33^. Due to the similarities between carotenoid and fat storage in fish skin, expression levels of lipid metabolism-related genes can indirectly reflect the content of carotenoids^39^. In our experiment, larvae at 1 dph had not yet commenced eating, and instead relied on their yolk sacs to provide nutrition. Therefore, upregulation of three lipid metabolism-related genes, namely *DGAT2* (diacylglycerol acyltransferase 2), *PNPLA2* (patatin-like phospholipase domain containing 2), and *APOD* (apolipoprotein D), as well as *SCARB1*, in YR45d and YR90d compared to YR1d, implies that carotenoids from the diet are stored, causing the metabolic rate to increase, which might be important for yellow pigment deposition in YR.

Another potentially important DEG is *BCO2* (beta-carotene oxygenase 2). As a pivotal gene responsible for carotenoid metabolism, *BCO2* is required for carotenoids to contribute to body color in tilapia (*Oreochromis niloticus*), and the expression level of this gene is important for converting colored carotenoids into colorless carotenoids^24,40^. In East African cichlid (*Tropheus duboisi*), higher expression of *BCO2* was observed in white areas adjacent to yellow skin, and similar results were reported for Chinook salmon (*Oncorhynchus tshawytscha*)^39,40^. Significant upregulation of *BCO2* in YR45d and YR90d might contribute to reduce the accumulation of colored carotenoids and maintain carotenoid homeostasis in skin.

Melanin is the most important pigment in animal skin, and can be divided into eumelanin (black or brown) and pheomelanin (yellow or red) based on differences in phenotype^41^. *SLC7A11*, also known as *xCT*, is a cysteine/glutamate exchanger that mediates the cellular uptake of cysteine (a component of pheomelanin) and thus directly affects pheomelanin synthesis^8^. In fish, many studies have shown a significant upregulation of *SLC7A11* in yellow skin samples compared with black samples^8,14^, indicating that expression differences in *SLC7A11* are strongly associated with yellow skin. In our current results, downregulation of *SLC7A11* expression was observed in YR45d and YR90d groups, and no other significant DEGs related to pheomelanin synthesis were identified, suggesting that the appearance of yellow skin in YR is probably caused by xanthophores rather than pheomelanin. Alternatively, the basal expression level of *SLC7A11* may be sufficient to induce the production of pheomelanin.

Interestingly, a series of genes significantly enriched in melanogenesis, the MAPK signaling pathway, and tyrosine metabolism were also upregulated in YR45d and YR90d, including tyrosinase gene family members, *MITF*, *MC1R*, *SLC45A2*, *OCA2*, *GPR143*, and *KITA*. Melanin biosynthesis in melanocytes is a complex biological process that involves a series of enzymatic reactions. l-Tyrosine and L-DOPA are recognized as the consecutive substrates and intermediates in melanogenesis, and tyrosinase (TYR), a key rate-limiting enzyme, can catalyze three different reactions in the melanogenic pathway; hydroxylation of monophenol (l-tyrosine), dehydrogenation of catechol (L-DOPA), and dehydrogenation of dihydroxyindole^42,43^. Other regulators in the melanogenesis pathway all play a role by activating or inhibiting the activity of TYR. For instance, Slominski et al.^43,44^ reported that α-melanocyte-stimulating hormone (α-MSH) binds to *MC1R*, resulting in up-regulation of cAMP and PKA levels, which in turn triggers eumelanin biosynthesis process by activating TYR. Although these genes are responsible for eumelanin synthesis, they were also identified in the skin of fish lacking melanophores, such as red tilapia (*Oreochromis* spp.)^45^, red crucial carp^14^, and Japanese ornamental carp^4^. In zebrafish, *MITF* and *MC1R* are involved in the formation of xanthophore lineages^46,47^. Our previous study also indicated that expression levels of *TYRP1a* and *TYRP2* were upregulated in YR in most periods from 1 dph to 12 months post-hatching compared to wild-type rainbow trout^48^, implying that these genes related to eumelanin synthesis might be involved in the formation of other pigment cells. Additionally, it has been reported that melanophores and xanthophores originate from the same precursor stem cells in zebrafish, and unstable xanthophores can be transformed into functional melanophores in amphibians^49,50^. Conversely, goldfish melanophores have also been confirmed to possess the ability to synthesize pteridine^51^. Consequently, higher expression of these genes might also be the result of melanophores transformed from xanthophores. Moreover, we also found that most of the genes discussed above, including pteridine/carotenoid/melanin synthesis-related genes, showed no significant differences between YR45d and YR90d groups, indicating that adult fish skin color might have basically formed at 45 dph.

## Conclusions

In this study, we performed a comparative analysis of skin transcriptomes during three pigmentation developmental stages in YR. A large number of DEGs related to pteridine/carotenoid/melanin synthesis were identified, which are possibly involved in the regulation of skin pigmentation. These candidate pigmentation-related genes were significantly enriched in key signaling pathways of pigment synthesis, such as melanogenesis, MAPK signaling, and tyrosine metabolism. In addition, 1,812,329 SSRs and 2,011,334 SNPs were detected in the YR skin transcriptome. The results improve our understanding of the molecular mechanism underlying skin pigmentation in YR, and could accelerate the molecular breeding of fish species with valuable skin color traits and will likely be highly informative for developing new therapeutic approaches to treat pigmentation disorders and melanoma.

## Materials and methods

### Experimental fish and sampling

**Figure 12:**
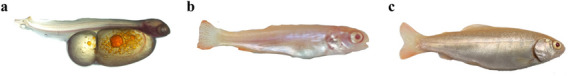
Three developmental stages of yellow mutant rainbow trout (YR). (**a**) 1 day post-hatching (YR1d). (**b**) YR45d. (**c**) YR90d.

Larvae and fry belonging to the full-sib YR family were collected from the Aquatic Science Training Center of Gansu Agricultural University, Gansu province, China, and these were cultured in a 3000 L cylindrical plastic water tank throughout the process from larvae to fry. The water temperature in the tank was maintained at 15 ± 1 °C, pH 7–8, dissolved oxygen (DO) = 9 ± 0.5 mg/L, and NH4-N < 0.1 mg/L. Fish were fed twice daily (9 a.m. and 3 p.m.) with commercial pellet feed. According to skin color changes during development, three developmental stages were determined: 1 dph, 45 dph, and 90 dph (Fig. 12). The number of individuals with a single biological replicate in each stage was altered according to size. At 1 dph, six larvae with heads and yolk sacs removed were pooled into one sample (n = 3) and denoted as YR1d1, YR1d2, and YR1d3. At 45 dph and 90 dph, dorsal skin of a single fry with scales removed was collected as one sample (n = 3) and denoted as YR45d1, YR45d2, YR45d3, YR90d1, YR90d2, and YR90d3. Fish were anesthetized with a lethal dose of MS-222 (50 mg/L; Sigma Aldrich Co., St. Louis, USA) before tissue sampling, and samples were collected immediately, flash-frozen in liquid nitrogen, and stored at − 80 °C. All experiments were performed according to the Guidelines for the Care and Use of Laboratory Animals in China, and the protocol was approved by the institutional ethic committee of Gansu Agricultural University (GSAU-Eth-AST-2021-004). The study was carried out in compliance with the ARRIVE guidelines.

### RNA isolation, library preparation, and sequencing

TRIzol reagent (Invitrogen, Carlsbad, CA, USA) was used to isolate nine RNA samples, and RNA quality was assessed by an Agilent 2100 Bioanalyzer (Agilent Technologies, Palo Alto, CA, USA) and checked using RNase-free agarose gel electrophoresis. Firstly, mRNAs were enriched by Oligo (dT) beads after total RNA was extracted. Secondly, mRNAs were fragmented into small pieces using fragmentation buffer and reverse-transcribed into cDNAs using random primers. Thirdly, second-strand cDNAs were synthesised with DNA polymerase I, RNase H, dNTPs, and buffer, and poly-A bases were added to the blunt ends of each strand to prepare them for ligation to the indexed adapters. Finally, paired-end sequencing was performed on an Illumina Novaseq6000 platform (Illumina, San Diego, CA, USA).

### Data preprocessing

All raw reads were cleaned before assembly, Fastp (version 0.18.0)^52^ was used to remove reads containing adapters, low-quality sequences, and unknown bases, and remaining clean reads were used for de novo assembly using Trinity 2.4.0^11^ with default settings. Resulting reads were then aligned to the rainbow trout reference genome (Omyk_1.1) using HISAT 2.2.4^53^ to identify gene expression and exon-exon splice junctions, and transcripts were constructed using Cufflinks (v2.2.1) and TopHat2 (v2.1.1)^54^. Transcript abundance was quantified by RSEM software with default settings, and a set of reference transcript sequences were obtained and preprocessed^55^. The FPKM value was used to calculate gene expression levels for each sample^4^.

### Screening of DEGs and analysis of temporal gene expression patterns

DEGs among the three compared groups were screened by the DESeq2 package (v.1.6.3) in R^56^. To ensure a high-quality analysis of DEGs, the FDR was used, and genes with a |log_2_ fold change| ≥ 1 and FDR < 0.05 were selected as DEGs.

To explore temporal differences in gene expression profiles, DEGs were subjected to trend analysis using STEM^54^. Each gene was assigned to the closest profile using a Pearson correlation-based distance metric. A permutation-based test was used to quantify the expected number of genes that would be assigned to each profile to determine the significance level of a given transcriptome profile^57^. Clustered profiles with p-values < 0.05 were defined as significant profiles.

### Functional annotation and PPI analysis of DEGs

GOseq software was used to annotate GO functions to reveal the biological functions of DEGs. Pathway annotations of DEGs were acquired using the KEGG database. GO enrichment analysis of DEGs was evaluated using the GOseq R package (Release 2.12)^58^. We used KOBAS v2.0 software to test the statistical enrichment of DEGs in KEGG pathway analysis^59^. The enrichment values of GO terms and KEGG pathways were calculated using hypergeometric tests, and DEGs with q-value < 0.05 were considered significantly enriched. Additionally, a PPI network of DEGs was constructed using the Search Tool for the Retrieval of Interacting Genes/Proteins (STRING, version 10.0) database^60^, which can clearly show interactions between proteins and whether gene-encoded proteins interact with each other. In the resulting network, nodes represent proteins and lines represent interactions between proteins. Cytoscape (version 3.4.0) software was applied to visualize the protein network.

### SSR and SNP identification

SSRs in the YR transcriptome were identified and analyzed by the MIcroSAtellite (MISA version 2.1) identification tool^61^. The parameters used to identify SSRs were at least six repeats for di-nucleotides and four repeats for tri-, tetra-, penta-, and hexa-nucleotides.

To identify putative SNPs in the transcripts, GATK (version 3.4-46)^62^ was used for calling variants of transcripts, and SNP annotation was conducted using ANNOVAR. The functions, genome sites, and types of variation of SNPs were also analyzed.

### Validation of gene expression by qRT-PCR

To validate the RNA-seq results, 14 DEGs with high levels of significance were selected and analyzed by qRT-PCR. All primers (Table 2) were designed using primer 5.0 software, and *β-actin* served as an internal control^63^. RNA samples that were used for the RNA-seq experiment were employed in this experiment. First-strand cDNAs were synthesised using a PrimerScript RT Reagent Kit with gDNA Eraser (Takara, Dalian, China). qRT-PCR was performed following the protocol provided with SYBR Premix Ex Taq (Takara, Dalian, China) on a LightCycler 480 II Instrument (Roche, Basel, Switzerland). The total volume of each amplification reaction was 20 μL, including 10 μL of SYBR Premix Ex Taq II (2×), 1 μL of each sense and antisense primer (10 μM), 0.5 μL of cDNA, and 7.5 μL of ddH_2_O. Thermal cycling was performed at 95 °C for 10 s, followed by 40 cycles at 95 °C for 5 s, and 60 °C for 20 s. Melting curve analyses was used to judge the specificity of the primers. The relative expression ratio of target genes versus *β-actin* was calculated using the 2^−∆∆Ct^ method^58^. Relative mRNA expression levels were statistically analyzed using SPSS 22.0 software.

## Supplementary Information

Supplementary Table S1.

Supplementary Table S2.

Supplementary Table S3.
